# Genetic and Phenotypic Characterization of the Novel Metallo-β-Lactamase NDM-29 From *Escherichia coli*

**DOI:** 10.3389/fmicb.2021.743981

**Published:** 2021-09-29

**Authors:** Ying Zhu, Xinmiao Jia, Peiyao Jia, Xue Li, Qiwen Yang

**Affiliations:** ^1^State Key Laboratory of Complex Severe and Rare Diseases, Department of Clinical Laboratory, Peking Union Medical College Hospital, Chinese Academy of Medical Sciences and Peking Union Medical College, Beijing, China; ^2^Graduate School, Peking Union Medical College, Chinese Academy of Medical Sciences, Beijing, China; ^3^State Key Laboratory of Complex Severe and Rare Diseases, Medical Research Center, Peking Union Medical College Hospital, Chinese Academy of Medical Sciences and Peking Union Medical College, Beijing, China; ^4^Department of Clinical Laboratory, Beijing Anzhen Hospital, Capital Medical University, Beijing, China

**Keywords:** carbapenemase, NDM, *Escherichia coli*, whole-genome sequence, antimicrobial resistance

## Abstract

**Objectives:** The New Delhi metallo-β-lactamase (NDM) can hydrolyze almost all clinically available β-lactam antibiotics and has widely spread all over the world. NDM-29, a novel carbapenemase, was discovered in an *Escherichia coli* (19NC225) isolated from a patient with biliary tract infection in 2019 in China.

**Methods:** Conjugation, transformation, cloning test, fitness cost, PacBio Sequel, and Illumina sequencing were performed to analyze the genetic and phenotypic characterization of *bla*_NDM–29_.

**Results:** The susceptibility testing results showed 19NC225 was resistant to cephalosporins, carbapenems, combinations of β-lactam and β-lactamase inhibitors, and levofloxacin. Conjugation and transformation were performed to verify the transferability of NDM-29-encoding plasmid, and cloning test was conducted to prove the function of *bla*_NDM–29_ to increase carbapenem resistance. Furthermore, fitness cost test confirmed that the presence of NDM-29 exerts no survival pressure on bacteria. PacBio Sequel and Illumina sequencing were performed to analyze the genetic characterization of 19NC225, which contains two plasmids (pNC225-TEM1B and pNC225-NDM-29). pNC225-NDM-29, exhibiting 99.96% identity and 100% coverage with pNDM-BTR (an IncN1 plasmid from an *E. coli* in urine specimen from Beijing in 2013), showed responsibility for the multidrug-resistant (MDR) phenotype. Compared with *bla*_NDM–1_, *bla*_NDM–29_, located on pNC225-NDM-29, carries a G388A (D130N) mutation. The region harboring *bla*_NDM–29_ is located in an ISKpn19-based transposon, and two Tn6292 remnants are symmetrically located upstream and downstream of the transposon. The sequence results also indicated several important virulence genes.

**Conclusion:** The findings of the novel carbapenemase NDM-29 could pose a threat to the control of antimicrobial resistance and arouse attention about the mutation of bacteria.

## Highlights

- The first detailed report of the carbapenemase NDM-29 from *E. coli* and its genetic environment in China.
- A whole genome sequence analysis of the newly found plasmid pNC225-NDM-29 and the gene *bla*_NDM–29_.
- Complete functional verification experiments, including conjugation, transformation, cloning, and fitness cost.

## Introduction

One popular resistant mechanism of gram-negative bacteria is producing hydrolytic enzymes, especially β-lactamases, which can hydrolyze β-lactam ring to break the amide bond and inactivate the antibacterial activity of drugs (Dever and Dermody, 1991). New Delhi metallo-β-lactamase (NDM) is classified as Ambler molecular class B β-lactamase, which can hydrolyze almost all clinically available β-lactam antibiotics (Khan et al., 2017; Liu et al., 2017). NDM-positive strains have widely spread all over the world, including India, Europe, China, Brazil, Australia, etc. (Khan et al., 2017; van Duin and Doi, 2017). Since NDM was first reported in a *Klebsiella pneumoniae* (*K. pneumoniae*) strain from a Swedish patient in New Delhi in 2009 (Yong et al., 2009), 31 variants of NDM (NDM-1–31) have been detected (NCBI) until now. Among them, NDM-1, 4, 5, and 7 are most prevalent around the world (Khan et al., 2017). NDM variant was discovered in not only human but also food animals and environment (van Duin and Doi, 2017; Liu et al., 2018). The highest distribution of NDM-positive species is observed in *K. pneumoniae* and *Escherichia coli* (*E. coli*) (Gamal et al., 2020).

In a recent study, we discovered an NDM-29 carbapenemase-producing *E. coli* strain (19NC225), isolated from a patient’s bile in 2019. The gene sequence of *bla*_NDM__–__29_ from a *Klebsiella pneumoniae* strain has been submitted to the NCBI database (NCBI), by researchers from Saint Petersburg, Russia (Starkova et al., 2021). Our study is the first detailed report of NDM—29 from *E. coli* in China, including genetic and phenotypic characterization, and confirms the potential threat of this new NDM-type carbapenemase to be a cause of extensively drug-resistant organism spread.

## Materials and Methods

### Bacterial Strains

The *E. coli* isolate 19NC225 was collected from the bile of a 65-year-old Chinese male patient with biliary tract infection in Nanchang, Jiangxi, China on March 2, 2019, and was identified by Vitek MS MALDI-TOF (BioMérieux) system in Peking Union Medical College Hospital.

*E. coli* DH5α, rifampin-resistant *E. coli* EC600, *K. pneumoniae* ATCC 13883, and *K. pneumoniae* ATCC 700603 were used in construction of transconjugants, transformants, and functional cloning experiments. The ampicillin-resistant pHSG575 plasmid was used in functional cloning experiments.

### Antimicrobial Susceptibility Testing

For donor isolates, recipient isolates, and transconjugants/transformants, the minimum inhibitory concentrations (MICs) of ertapenem, cefoxitin, imipenem, ceftriaxone, piperacillin/tazobactam, ceftazidime, ceftazidime/avibactam, meropenem, ceftolozane/tazobactam, cefepime, amikacin, levofloxacin, aztreonam, and imipenem-relebactam were determined by broth microdilution method according to CLSI M100 documents (Institute Clinical and Laboratory Standards, 2015). The MICs were measured only when the quality controls were acceptable. *E. coli* ATCC25922 and *Pseudomonas aeruginosa* ATCC27853 were used as quality controls.

### Conjugation

The transferability of pNC225-NDM-29 among isolates was determined using 19NC225 as donor and *E. coli* DH5α, *E. coli* EC600, *K. pneumoniae* ATCC 13883, and *K. pneumoniae* ATCC 700603 as the recipients. The procedure of conjugation was performed according to the protocol previously described (Yang, 2015). Into 40 ml antibiotic-free Luria–Bertani (LB) broth, 100 μl overnight culture of clinical isolate (19NC255) in LB broth containing meropenem (2 μg/ml) and 200 μl overnight culture of recipients DH5α, EC600, ATCC 13883, or ATCC 700603 in LB broth containing rifampin (100 μg/ml) were mixed and were incubated at 37°C for 18 h. Then, the conjugant mixtures were placed on selective LB agar containing 100 μg/ml rifampin and 2 μg/ml meropenem. PCR analysis and agarose gel electrophoresis were performed to confirm the effects of transconjugation.

### Transformation

Transformation was performed through electrophoration. Competent cells of recipients for electrophoration were prepared according to the procedure previously described (Wang R. et al., 2018; Wang Y. et al., 2018). Overnight cultures of recipients (*E. coli* DH5α, *K. pneumoniae* ATCC 13883, and *K. pneumoniae* ATCC 700603) in LB broth were diluted 1:100 into 100 ml LB broth and were incubated at 37°C. The culture was immediately cooled on ice for 20 min once the optical density at 600 nm (OD600) of the cell culture reached 0.5–0.7. Then, the culture was centrifuged at 4,500 rpm for 5 min. The supernatant was discarded, and 15 ml of sterile ice-cold 10% glycerol was pipetted into the cells. The mixture was resuspended gently. The centrifugation and pipetting steps were repeated thrice in all. Finally, the supernatant was discarded, and the cells were resuspended with 1 ml of ice-cold 10% glycerol. All cell-preparing procedures were performed on ice. Competent cells should be stored at –80°C for long-term use.

Electroporation was also conducted according to the procedure previously described (Wang R. et al., 2018; Wang Y. et al., 2018). Fifty microliters of electrocompetent cells, which have been thawed on ice, were mixed with no more than 5 μl plasmid. The mixture was transferred to a prechilled, sterile electroporation cuvette (0.2 cm) and placed in the electroporation apparatus (Bio-Rad Laboratories, Richmond, CA) (parameter setting: 2.5 kV, 200 Ω, and 25 μF). After being pulsed, the cells were recovered in 1 ml antibiotic-free LB broth and incubated at 37°C for 1.5 h before being selected by LB agar plates containing meropenem (2 mg/l). Then, the plates were incubated at 37°C overnight.

### Functional Cloning of NDM-29

To confirm the drug-resistant phenotypes conferred by the suspected resistance gene, *bla*_NDM–29_ and *bla*_NDM–1_ were ligated into the α-complementation plasmid vector pHSG575 (Takeshita et al., 1987). The recombinant plasmid was introduced into *E. coli* DH5α, *E. coli* EC600, *K. pneumoniae* ATCC 13883, and *K. pneumoniae* ATCC 700603 by electroporation as described before. The transformants were selected on LB agar containing 50 mg/l chloramphenicol, and PCR was conducted to confirm the presence of plasmid pHSG575-NDM-29. Subsequently, antimicrobial susceptibility testing was performed to determine the microbiology phenotype of the transformants. Primers used in the experiments are listed in Supplementary Table 1.

### Fitness Cost

Growth curve was used to assess the fitness impact of plasmid carriage under non-competitive conditions as Wang R. et al., 2018 described previously. The recipients and transconjugants/transformants carrying pNC225/NDM-29 plasmid from 19NC225 isolate were cultured overnight in LB broth containing 2 mg/l meropenem at 37°C. Bacterial suspensions were diluted to a 0.5 McFarland standard and then diluted 1:100 in LB medium (approximately 10^6^ CFU/ml) and grown at 37°C in triplicate for 48 h by Epoch^TM^ 2 Microplate Spectrophotometer from BioTek Instruments. The OD600 of each culture was measured every 30 min, and the plates were shaken for 15 s before measuring. The growth curves were estimated by GraphPad Prism version 8 (GraphPad Software, Inc., United States). Statistical significance was defined for an overall error at 0.05 level (95% confidence interval) using one-way analysis of variance (ANOVA) followed by Tukey tests.

### Genomic DNA Extraction, Sequencing, Assembly, Correction, and Annotation

Genomic DNA was extracted using UltraClean^®^ Microbial DNA Isolation Kit (MOBIO Laboratories, Inc.). Whole-genome sequencing was implemented using the PacBio Sequel platform. A 20-kb SMRTbell library was prepared from sheared genomic DNA (≥5 g) with an additional bead clean-up step before primer annealing (Zhu et al., 2016).

To correct the polymer errors produced during PacBio sequencing, we re-sequenced this isolate using next-generation sequencing. Paired-end libraries were prepared from 5 μg of isolated genomic DNA using a TruSeq DNA sample prep kit (Illumina Inc., San Diego, California, United States) and sequenced with a read length of 2 × 150 nucleotides using an Illumina Genome Analyzer IIx. Raw Illumina sequencing reads were trimmed at a threshold of 0.01 (Phred score of 20). *De novo* assembly of the genome was performed using Unicycler (Wick et al., 2017) from short and long sequencing reads.

Genome sequences were annotated with the rapid prokaryotic genome annotation software Prokka (Seemann, 2014). For the assembled plasmids, blastn was first carried out to search the reference plasmids according to the similarity of sequence. The plasmids and its references were submitted to RAST, Glimmer, GeneMarkS, and prodigal for gene prediction, after finding replication origin (repR or repA). The plasmids with rough annotation from Prokka (Seemann, 2014) were added with detailed comments on its CDS and MGE using NCBI, ExPASy, ISFinder, and INTEGRALL. Then, the genome structure of plasmids was compared to analyze their evolutionary relationships. The genome structure comparisons among plasmids were performed to analyze the sequence homology. Plasmid circular structure maps were generated with BRIG software.

### Virulence Genes and Antimicrobial Resistance Genes Analysis

Virulence genes were downloaded from virulence factor database (VFDB). FASTA sequences of these genes were used to search for corresponding genes by using BLAST with a coverage of 50% and identity of 90%. Antimicrobial resistance genes were identified using ResFinder 4.0 with the whole genomic sequences.

### Sequence Type, Serotype, and Phylogenetic Analysis

Sequence types (STs) were determined using SRST2 (Inouye et al., 2014) based on the Illumina reads. Serotype was analyzed using SerotypeFinder 2.0 (Joensen et al., 2015) based on the assembled contigs. In order to obtain the phylogenetic features of 19NC225, the genome sequences of 99 *E. coli* strains containing *bla*_NDM_ gene were downloaded from NCBI. The phylogenetic analysis of 19NC225 and 99 downloaded strains was based on the core-genome SNPs detected by MUMmer 3.23 using the assembled genome of 19NC225 (NC_000962) as the reference (Delcher et al., 2002). The MAFFT (Nakamura et al., 2018) was adopted to align the concatenated SNP sequences, and phylogenetic tree was generated by FastTree (Price et al., 2009).

### Nucleotide Sequence Accession Numbers

The genome data have been deposited in NCBI with the GenBank accession number CP066844.

## Results

### Clinical Information

The clinical isolate 19NC225 was an *E. coli* strain isolated from bile of a 65-year-old Chinese male patient with biliary tract infection in Nanchang, Jiangxi, China collected on March 2, 2019. The patient was hospitalized with hepatolithiasis coupled with cholangitis and had a history of diabetes, hypoproteinemia, incursion treatment, operation, and antibiotic use. In the duration of hospitalization, the patient was treated with cefoperazone/sulbactam (3 g, q 12 h, February 19, 2019–February 27, 2019), ornidazole (0.75 g, q 12 h, March 4, 2019–March 5, 2019), and deoxycephalosporin (1 g, q 12 h, March 4, 2019–March 5, 2019). Antimicrobial chemotherapy did not take effect. The pathogen still existed in bile after therapy, and the patient was uncured when discharged from hospital (March 12, 2019).

The susceptibility testing results showed that the clinical isolate 19NC225 was resistant to almost all β-lactams examined, including cephalosporins (cefoxitin, ceftriaxone, ceftazidime, and cefepime), carbapenems (ertapenem, imipenem, and meropenem), combinations of β-lactams and β-lactamase inhibitors (piperacillin/tazobactam, ceftazidime/avibactam, ceftolozane/tazobactam, and imipenem-relebactam), and levofloxacin. The strain was only susceptible to aztreonam and amikacin (Table 1).

**TABLE 1:** MIC of the clinical strain, donors, and recipients with/without pNC225/NDM-29.

Strain	19NC225	EC600	TfEC600^a^	TcEC600^b^	DH5α	TfDH5α^a^	13883	Tf13883^a^	Tc13883^b^	700603	Tf700603^a^	Tc700603^b^
Ertapenem	>4	≤0.06	>4	>4	≤0.06	>4	≤0.06	>4	>4	≤0.06	>4	>4
Cefoxitin	>16	≤2	>16	>16	8	>16	8	>16	>16	>16	>16	>16
Imipenem	>16	0.25	>16	16	0.25	>16	0.5	>16	>16	0.25	>16	>16
Ceftriaxone	>8	≤0.5	>8	>8	≤0.5	>8	≤0.5	>8	>8	4	>8	>8
Piperacillin/tazobactam	>64/4	≤2/4	>64/4	>64/4	≤2/4	>64/4	≤2/4	>64/4	>64/4	>64/4	>64/4	>64/4
Ceftazidime	>16	≤0.5	>16	>16	≤0.5	>16	≤0.5	>16	>16	>16	>16	>16
Ceftazidime/avibactam	>16/4	0.5/4	>16/4	>16/4	≤0.06/4	>16/4	0.25/4	>16/4	>16/4	0.5/4	>16/4	>16/4
Meropenem	>16	≤0.06	>16	>16	≤0.06	>16	≤0.06	>16	>16	≤0.06	>16	>16
Ceftolozane/tazobactam	>16/4	1/4	>16/4	>16/4	≤0.06/4	>16/4	0.5/4	>16/4	>16/4	2/4	>16/4	>16/4
Cefepime	>16	≤0.5	>16	>16	≤0.5	>16	≤0.5	>16	>16	≤0.5	>16	>16
Amikacin	≤4	≤4	≤4	≤4	≤4	≤4	≤4	≤4	≤4	≤4	≤4	≤4
Levofloxacin	>4	≤0.25	>4	4	≤0.25	2	≤0.25	2	2	1	>4	>4
Aztreonam	≤0.5	≤0.5	≤0.5	≤0.5	≤0.5	≤0.5	≤0.5	≤0.5	≤0.5	>8	>8	>8
Imipenem-relebactam	>16/4	0.5/4	>16/4	8/4	0.25/4	>16/4	2/4	>16/4	>16/4	0.25/4	>16/4	>16/4

*^a^Tf, transformants of recipient.*

*^b^Tc, transconjugants of recipient.*

### Conjugation and Transformation of the Resistance Plasmid (pNC225-NDM-29)

In the study, *E. coli* DH5α, *E. coli* EC600, *K. pneumoniae* ATCC 13883, and *K. pneumoniae* ATCC 700603 were used for conjugation of plasmids in 19NC225. Due to the presence of two plasmids (pNC225-NDM-29 and pNC225-TEM1B) in donor 19NC225, we performed agarose gel electrophoresis and PCR to confirm that all the conjugants (*E. coli* EC600, *K. pneumoniae* ATCC 13883, and *K. pneumoniae* ATCC 700603) only obtained NDM-29 gene without TEM-1 gene. It confirmed that the plasmid harboring *bla*_NDM–29_ in the clinical strain 19NC225 has the potential for horizontal transfer. Considering the low efficiency of the transconjugation of *K. pneumoniae* ATCC 13883 and *K. pneumoniae* ATCC 700603, the transformants of ATCC 13883 and ATCC 700603 were constructed through electroporation. The transformants of DH5α are also constructed by electroporation. As shown in Table 1, the antimicrobial susceptibility of transformants was similar with that of corresponding transconjugants. Compared to the plasmid-free counterparts, plasmid pNC225-NDM-29 reduced the susceptibility of transconjugants/transformants to almost all β-lactams examined. Except for ATCC 700603 that was resistant to cefoxitin, ceftriaxone, ceftazidime, piperacillin/tazobactam, and aztreonam naturally, the susceptibility results of all transformants were consistent with the donor (clinical isolate 19NC225): resistant to cephalosporins, carbapenems, combinations of penicillin and β-lactamase inhibitors, and levofloxacin.

### Cloning Test for *bla*_NDM–29_

To determine the specific function of carbapenemase NDM-29, the genes *bla*_NDM–29_ and *bla*_NDM–1_ were cloned into pHSG575 (pHSG575/NDM-29 and pHSG575/NDM-1), and the corresponding transconjugants/transformants were detected to determine their antimicrobial susceptibility. As shown in Table 2, compared to the transformants with the plasmid pHSG575, transformants with plasmid pHSG575-NDM-29 showed higher resistance to antibiotics including cephalosporins, carbapenems, and combinations of penicillin and β-lactamase inhibitors. The transformants with plasmid pHSG575/NDM-29 showed no difference in susceptibility to the drugs detected with the transformants with plasmid pHSG575/NDM-1. It is worth noticing that transformants with cloned plasmid pHSG575/NDM-29 maintained the susceptibility to levofloxacin, while transformants with plasmid pNC225/NDM-29 showed an increased resistance to levofloxacin, which suggested that the drug resistance gene for levofloxacin is located on the plasmid pNC225-NDM-29, which was in accordance with the WGS analysis as the gene *qnrS1* can imply quinolone resistance.

**TABLE 2:** MIC of different transformants with pHSG575/NDM-29 and the recipients.

Strain	EC600	EC600 + NDM-1	EC600 + NDM-29	DH5a	DH5a + NDM-1	DH5a + NDM-29	13883	13883 + NDM-1	13883 + NDM-29	700603	700603 + NDM-1	700603 + NDM-29
Ertapenem	≤0.06	>4	>4	≤0.06	>4	>4	≤0.06	>4	>4	≤0.06	>4	>4
Cefoxitin	8	>16	>16	≤2	>16	>16	≤2	>16	>16	16	>16	>16
Imipenem	0.25	>16	8	0.25	16	>16	0.5	>16	>16	0.25	>16	>16
Ceftriaxone	≤0.5	>8	>8	≤0.5	>8	>8	≤0.5	>8	>8	4	>8	>8
Piperacillin/tazobactam	≤2/4	>64/4	>64/4	≤2/4	>64/4	>64/4	≤2/4	>64/4	>64/4	>64/4	>64/4	>64/4
Ceftazidime	≤0.5	>16	>16	≤0.5	>16	>16	≤0.5	>16	>16	>16	>16	>16
Ceftazidime/avibactam	0.25/4	>16/4	8/4	≤0.06/4	>16/4	>16/4	0.25/4	>16/4	>16/4	0.5/4	>16/4	>16/4
Meropenem	≤0.06	>16	16	≤0.06	16	>16	≤0.06	>16	>16	≤0.06	>16	>16
Ceftolozane/tazobactam	0.5/4	>16/4	>16/4	≤0.06/4	>16/4	>16/4	0.5/4	>16/4	>16/4	1/4	>16/4	>16/4
Cefepime	≤0.5	>16	>16	≤0.5	>16	>16	≤0.5	>16	>16	≤0.5	>16	>16
Amikacin	≤4	≤4	≤4	≤4	≤4	≤4	≤4	≤4	≤4	≤4	≤4	≤4
Levofloxacin	≤0.25	≤0.25	≤0.25	≤0.25	≤0.25	≤0.25	≤0.25	≤0.25	≤0.25	1	1	1
Aztreonam	≤0.5	≤0.5	≤0.5	≤0.5	≤0.5	≤0.5	≤0.5	≤0.5	≤0.5	>8	>8	>8
Imipenem-relebactam	0.25/4	>16/4	8/4	0.25/4	>16/4	>16/4	1/4	>16/4	>16/4	≤0.06/4	>16/4	>16/4

### Fitness Cost

**FIGURE 1:**
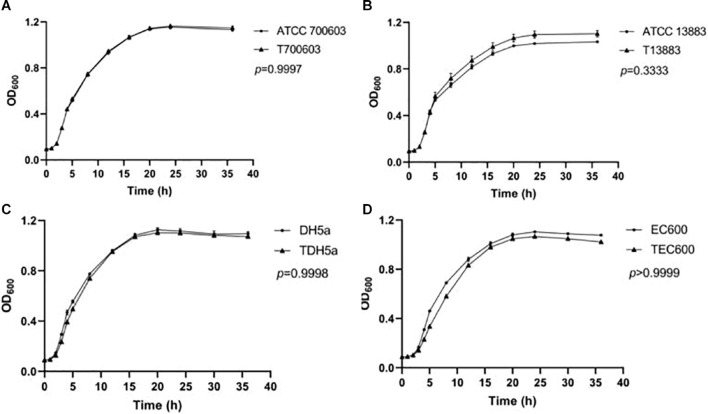
Growth curve of transformants containing pNC225-NDM-29. One-way analysis of variance (ANOVA) followed by Tukey tests was used to define the statistical significance. *P* > 0.05 indicates no fitness cost was observed within pairs. **(A)** Growth curve of ATCC 700603 and corresponding transformant containing pNC225-NDM-29. **(B)** Growth curve of ATCC 13883 and corresponding transformant containing pNC225-NDM-29. **(C)** Growth curve of DH5a and corresponding transformant containing pNC225-NDM-29. **(D)** Growth curve of EG600 and corresponding transformant containing pNC225-NDM-29. T700603, transformant of ATCC 700603; T13883, transformant of ATCC 13883; TDH5α, transformant of DH5α; TEC600, transformant of EC600.

To future analyze the impact of the plasmid to the survival of strains, fitness cost assay was conducted, and growth curve is shown in Figure 1. The growth rates of transconjugants/transformants showed no difference compared to their donor strains. No significant difference in growth rates was found within pairs (*p* > 0.05). Therefore, the antibiotics resistance mediated by pNC225-NDM-29 did not increase the growth fitness cost of the resistant strains compared to its susceptible counterparts.

### Genomic Characteristics and Antimicrobial-Resistance Genotype Analysis

**FIGURE 2:**
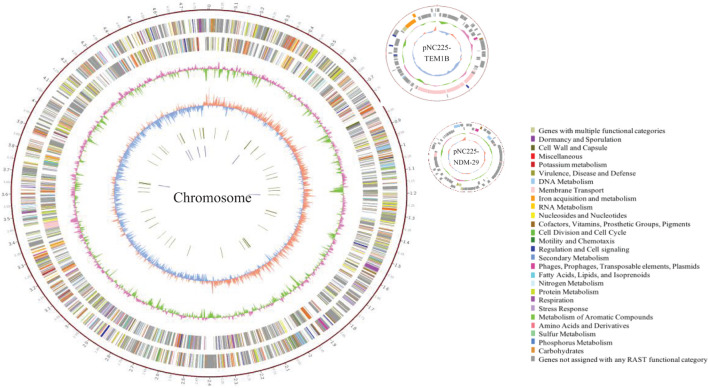
Circular representation of 19NC225 genome (chromosome and two plasmids). The complete genome of the clinical isolate 19NC225 includes a 4,804,173-bp chromosome, a 127,534-bp plasmid (pNC225-TEM1B), and a 60,935-bp plasmid (pNC225-NDM-29). Circles are shown as follows (outside to inside): (1) A physical map scaled in megabases (Mb) from base 1 (the start of the putative replication origin); (2) coding sequences transcribed in a clockwise direction; (3) coding sequences transcribed in a counter-clockwise direction; (4) G + C content; (5) GC skew; (6) tRNA genes; and (7) rRNA genes. Genes displayed in (2) and (3) are color-coded according to different functional categories shown on the right.

**FIGURE 3:**
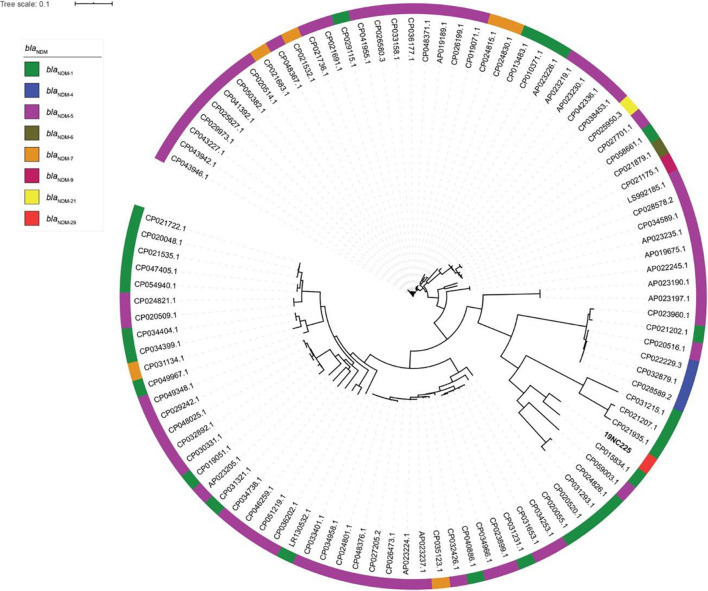
Phylogenic tree of *E. coli* strains with complete genomes containing *bla*_NDM_ variants. The colored strips on the outside ring indicate the eight *bla*_NDM_ variants.

To reveal the genetic basis of the multidrug-resistant (MDR) phenotype, we obtained the complete genome of the clinical isolate 19NC225 including a 4.8-Mb chromosome, a 128-kb plasmid (pNC225-TEM1B), and a 60-kb plasmid (pNC225-NDM-29) (Table 3 and Figure 2). Bioinformatics analysis provided the general chromosome genome information, including GC% content (50.63%), and predicted protein coding genes (4,576), average gene length (938 bp), and coding region (89.39%). The two plasmids possess a higher GC content (∼52%), lower ratio of coding regions, and shorter average gene length. The phylogenetic analysis (Figure 3) showed that 19NC225, belonging to ST1485 and O83:H42, is clustered together with *E. coli* strain MS6198 from Australia (Hancock et al., 2017), which contains a *bla*_NDM–1_-positive IncA/C plasmid.

**TABLE 3:** The genomic characteristics and antimicrobial resistance genotype analysis of the sequences of chromosomes and plasmids of 19NC225.

Name*	Genome size (bp)	GC content	Coding genes	Average gene size (bp)	Coding region (bp)	tRNA	rRNA	Drug resistance genes
NC225-chr	4,804,173	50.63%	4,576	938	4,294,518 (89.39%)	86	22	*mdfA*
pNC225-TEM1B	127,534	51.93%	148	743	110,007 (86.25%)	0	0	*tetA*, *aph(3”)-Ib*, *aph(6)-Id*, *dfrA14*, *bla_TEM–1B_*, and *sul2*
pNC225-NDM-29	60,935	51.79%	85	589	50,070 (82.17%)	0	0	*dfrA14*, *qnrS1*, *bla_NDM–29_*, and *ble*_MBL_

*Sequences of chromosomes end with the word “chr;” sequences of plasmids begin with the word “p.”

Virulence genes (Supplementary Table 2) analysis showed that the clinical isolate 19NC225 contains 235 virulence genes, 12 of which are located on the 128-kb plasmid (pNC225-TEM1B), including aerobactin synthesis and transport protein (*iutA*, *iucABCD*, and *ECVR50_3327*), ferrous iron transport proteins (*sitABCD*), and *aaiQR*. No virulence genes were found on the 60-kb plasmid (pNC225-NDM-29).

Nine categories of antimicrobial-resistance genes were discovered in 19NC225 including one antimicrobial resistance gene on the chromosome: multidrug transporter *mdfA*, an efflux pump driven by the proton motive force that confers resistance to a broad spectrum of chemically unrelated drugs; six antimicrobial resistance genes on the 128-kb plasmid (pNC225-TEM1B): a tetracycline resistance gene (*tetA*), two aminoglycoside resistance genes [*aph(3″)-Ib* and *aph(6)-Id*], one sulfonamide resistance gene (*sul2*), one trimethoprim resistance gene (*dfrA14*), and one β-lactamase gene (*bla*_TEM–1B_); four antimicrobial resistance genes on the 60-kb plasmid (pNC225-NDM-29): one quinolone resistance gene (*qnrS1*), one trimethoprim resistance gene (*dfrA14*), one bleomycin resistance gene (*ble*MBL), and importantly one carbapenemase resistance gene *bla*_NDM–29_ (a very rare subtype of *bla*_NDM,_ that will be discussed in detail in subsequent sections, Table 2). These antimicrobial-resistance genes, especially *bla*_NDM–29_, are responsible for the MDR phenotype, including most β-lactam drugs, which is in accordance with antimicrobial susceptibility testing.

### Identification of the Novel *bla*_NDM–29_ Carbapenemase and Characterization of the *bla*_NDM–29_ Harboring Plasmid pNC225-NDM-29

Antimicrobial-resistance genes analysis showed a *bla*_NDM_ gene located on the 60-kb plasmid (pNC225-NDM-29). Sequence alignment presented that this *bla*_NDM_ gene shares 100% identity with *bla*_NDM–29_ [carrying a G388A (D130N) mutation compared to *bla*_NDM–1_] (Supplementary Figure 1), a very rare subtype of *bla*_NDM_ found in *K. pneumoniae*. This is the first report of this subtype in *E. coli* in the world.

**FIGURE 4:**
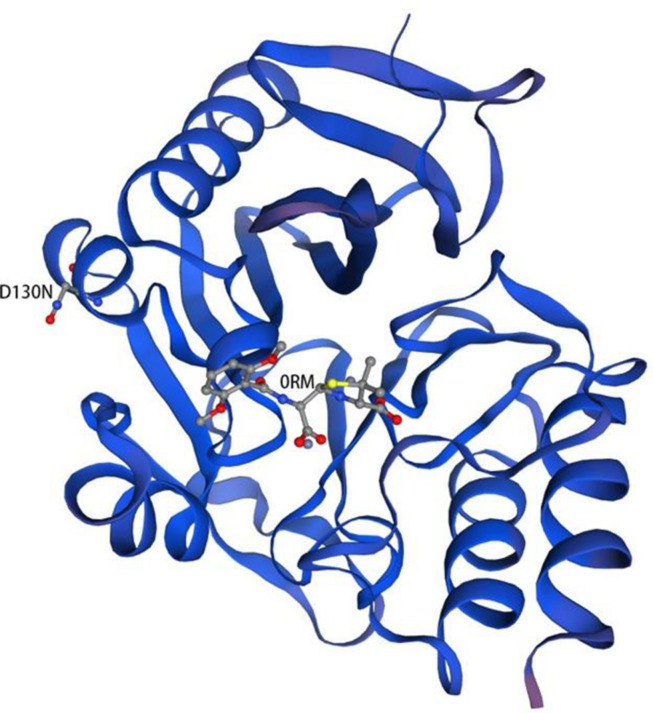
Homology model of NDM-29. Protein backbone of NDM-29 was shown with the helices and strands. 0RM ((2R,4S)-2-{(R)-carboxy[(2,6-dimethoxybenzoyl)amino]methyl}-5,5-dimethyl-1,3-thiazolidine-4-carboxylic acid) was bound to NDM-29 protein. The mutation (D130N) was labeled on the left helix (Guex et al., 2009; Bertoni et al., 2017; Bienert et al., 2017; Waterhouse et al., 2018; Studer et al., 2020).

The predicted model of NDM-29 was generated by SWISS-MODEL according to homology modeling using NDM-1 as a template (PDB accession no. 4EY2) (Figure 4). There is a metal binding at the position 124, which is near the mutation site (D130N, Berman et al., 2000).

The complete nucleotide sequence of the plasmid pNC225-NDM-29 carrying *bla*_NDM–29_ was 60,935 bp in length, constituting a circular DNA with an average G + C content of 51.79%. Eighty-five open reading frames were annotated (Table 2) because it contained an IncN-type *repA* (plasmid replication initiation) gene (D130N).

**FIGURE 5:**
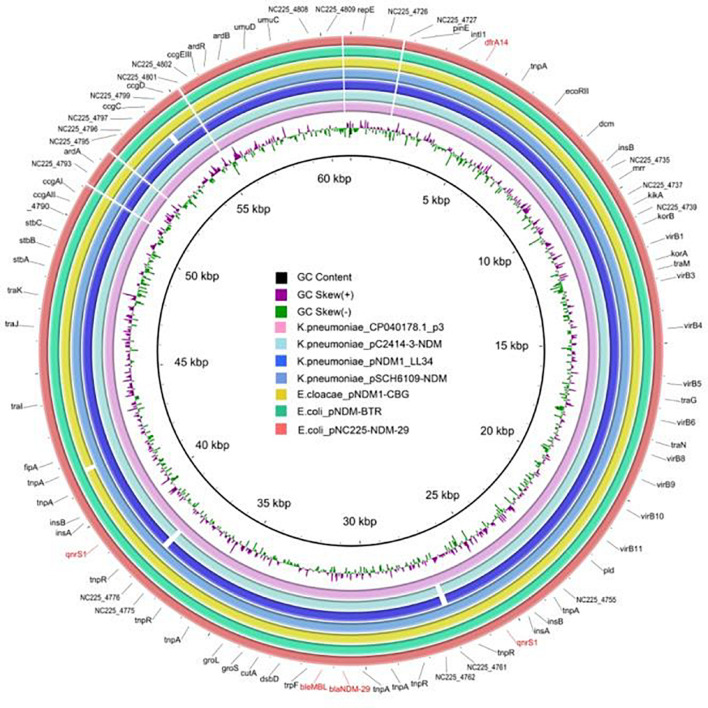
Genetic structure of pNC225-NDM-29 and six *bla*_NDM–1_-harboring plasmids (pSCH6109-NDM, pNDM1_LL34, pC2414-3-NDM, and CP040178.1_p3 from *Klebsiella pneumoniae*; pNDM1-CBG from *Enterobacter cloacae*; and pNDM-BTR from *Escherichia coli*). Alignments of resemble plasmids are shown as concentric rings. The outermost shows the main coding genes of pNC225-NDM-29. Antimicrobial-resistance genes are highlighted in red.

**FIGURE 6:**
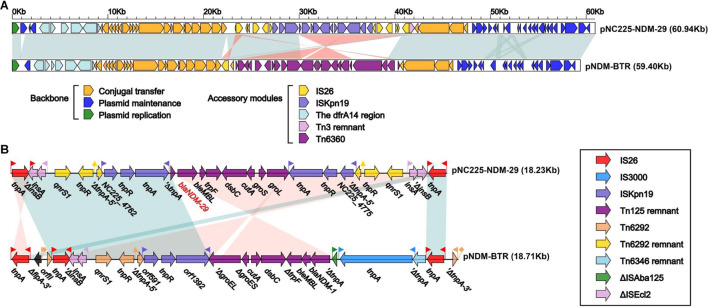
Structure of pNC225-NDM-29 and related transposon. **(A)** Linear comparison of plasmid pNC225-NDM-29 with the most similar plasmid pNDM-BTR. Genes are denoted by arrows. Genes, mobile elements, and other features are colored based on functional classification. Shading denotes the regions with high homology (95% nucleotide identity). **(B)** The linear genomic comparison of the drug resistance region in plasmids between pNC225-NDM-29 and pNDM-BTR. The whole region containing main functional areas of pNC225-NDM-29 is located inside two opposite-directed IS26 elements, formatting an IS26-based transposon structured as IS16-ΔISEcl2-qnrS1-Tn6292 remnant-ISKpn19-*bla*_NDM–29_-bleMBL-trpF-dsbC-cutA-groS-groL-ISKpn19-Tn6292-remnant-qnrS1-ΔISEcl2-IS16.

pNC225-NDM-29 was assigned to the IncN group. We further performed a full-plasmid BLAST comparative analysis. The result showed that pNC225-NDM-29 exhibited 99% identity with six *bla*_NDM_ containing plasmids: four plasmids of *K. pneumoniae* (pSCH6109-NDM, pNDM1_LL34, pC2414-3-NDM, and CP040178.1_p3), one plasmid of *Enterobacter cloacae* (pNDM1-CBG), and one plasmid of *E. coli* (pNDM-BTR), with high coverages (99, 99, 99,100, 99, and 100%, respectively, Figure 5). These six plasmids are *bla*_NDM–1_-carrying ones. A linear genomic comparison was further conducted between pNC225-NDM-29 and pNDM-BTR (*bla*_NDM–1_-carrying plasmid) in *E. coli* (Figure 6A) since they share high identity and both belong to *E. coli*. The backbone region and accessory modules containing *dfrA14* show high similarity to that of pNDM-BTR with 99.96% identity and 100% coverage, indicating the high-level conservation of this IncN backbone. However, there is an inversion of the accessory modules containing *bla*_NDM–29_ compared with the *bla*_NDM–1_ region of pNDM-BTR. As shown in Figure 6B, the region harboring *bla*_NDM–29_ is located in an ISKpn19-based transposon, with two ISKpn19 elements at terminal regions (in opposite directions). This region also contained a bleomycin resistance protein (bleMBL), trpF, dsbC, cutA, groS, and groL. Additionally, two Tn6292 remnants, carrying quinolone resistance gene *qnrS1*, are symmetrically located upstream and downstream of this ISKpn19-based transposon. Moreover, the whole region containing the above elements is located inside two opposite-directed IS26 elements. This provided evidence of insertion of this region into the backbone of the plasmid, based on IS26- or ISkpn19-mediated intermolecular replicative transposition.

## Discussion

Here we described a newly found carbapenemase, NDM-29, isolated from a clinical strain of *E. coli*. The success of conjugation from clinical isolation to *E. coli* EC600, *K. pneumoniae* ATCC 13883, and *K. pneumoniae* ATCC 700603 indicated the transferability of pNC225-NDM-29. The presence of the ISKpn19-based transposon where the region harboring *bla*_NDM__–__29_ is located suggested the risk of the spread of resistance caused by NDM-29. Meanwhile, no fitness cost was observed in the transconjugants/transformants containing plasmid pNC225-NDM-29, which demonstrated a limited burden from the transferable plasmid. However, the conjugation from donor to recipients (ATCC 13883, ATCC 700603, and DH5α) showed low efficiency, which may be due to high energy cost or multibarrier in recipients (Llosa et al., 2002). Although the growth curves did not show a significant impact of transferred plasmid on recipients, a limitation of growth curve that the competitive fitness of donor over recipients was not estimated and needs further study (Hanafi et al., 2016). Anyhow, the dissemination and adaptability of the NDM-29-harboring plasmid among clinical bacteria would be a threat to infectious control.

The pNC225-NDM-29 shows high identity with six NDM-1-carrying plasmids (≥ 99%). The highest identity (99.96%) appears between pNC225-NDM-29 and pNDM-BTR, an NDM-1-encoding plasmid from an *E. coli* strain BTR isolated from the urine specimen of an 89-year-old female patient with chronic obstructive pulmonary disease in Beijing in 2013 (Zhao et al., 2017). Considering the transposon near NDM-29, we tend to believe NDM-29 is likely a mutation of NDM-1 produced during propagation. This suggests some genomic functions such as high transmission capacity and pathogenicity, and influence on patient prognosis of *bla*_NDM–29_ may be similar to *bla*_NDM–1_. Whether the mutation will cause the change of carbapenem resistance needs further validation. The prevalence of NDM-29 existing in animals also needs more surveillance and research.

Currently, the mutation of D130G has been reported in NDM-14, and kinetic analysis indicated that NDM-14 has greater carbapenem resistance with a higher affinity for imipenem and meropenem (Zou et al., 2015). Based on our research, there is no difference between NDM-1 and NDM-29 in terms of susceptibility to carbapenems, which is in accordance with the findings of Starkova et al. (2021).

In conclusion, we report the identification of a novel class B enzyme with carbapenemase activity, NDM-29, in a clinical *E. coli* isolate. The novel *bla*_NDM__–__29_ is first detected in China and was obtained from a MDR *E. coli* strain isolated from bile of a patient with biliary tract infection. The strain, containing two plasmids (pNC225-TEM1B and pNC225-NDM-29), belongs to ST1485 and O83:H42, showed homology with *E. coli* MS6198 from Australian (Hancock et al., 2017), which harbors *bla*_NDM__–__1_. The plasmid, pNC225-NDM-29, which encods *bla*_NDM__–__29_, exhibited 99% identity with six *bla*_NDM__–__1_-carrying plasmids, especially a IncN1 plasmid pNDM-BTR from an *E. coli* in urine specimen (99.96% identity and 100% coverage), and showed responsibility for the MDR phenotype.

Recently, the reports of new β-lactamase genes have increased, especially those with carbapenemase activity. Because of the travel of infected or colonized individuals between countries, NDM-1 became a global epidemic in fewer than 5 years since the first discovery (Bush and Bradford, 2020). With more and more NDM variants found in animals, we further need to attach great importance to the threat of antimicrobial resistance from food. Thus, the research of the original newly discovered variants is valuable for surveillance of resistance outbreak and the genetic evolution of bacteria.

## Data Availability Statement

The datasets presented in this study can be found in online repositories. The names of the repository/repositories and accession number(s) can be found below: https://www.ncbi.nlm.nih.gov/genbank/, CP066844.

## Ethics Statement

The Human Research Ethics Committee of Peking Union Medical College Hospital approved this study and waived the need for consent (Ethics Approval Number: S-K238).

## Author Contributions

YZ and XJ wrote the manuscript. PJ, XL, and QY revised the manuscript. YZ, XJ, PJ, and XL performed the experiments. QY conceived and designed the study. All authors contributed to the article and approved the submitted version.

## Conflict of Interest

The authors declare that the research was conducted in the absence of any commercial or financial relationships that could be construed as a potential conflict of interest.

## Publisher’s Note

All claims expressed in this article are solely those of the authors and do not necessarily represent those of their affiliated organizations, or those of the publisher, the editors and the reviewers. Any product that may be evaluated in this article, or claim that may be made by its manufacturer, is not guaranteed or endorsed by the publisher.
