# Corrigendum: Genetic and Phenotypic Characterization of the Novel Metallo-β-Lactamase NDM-29 From *Escherichia coli*

**DOI:** 10.3389/fmicb.2021.795790

**Published:** 2021-11-09

**Authors:** Ying Zhu, Xinmiao Jia, Peiyao Jia, Xue Li, Qiwen Yang

**Affiliations:** ^1^State Key Laboratory of Complex Severe and Rare Diseases, Department of Clinical Laboratory, Peking Union Medical College Hospital, Chinese Academy of Medical Sciences and Peking Union Medical College, Beijing, China; ^2^Graduate School, Peking Union Medical College, Chinese Academy of Medical Sciences, Beijing, China; ^3^State Key Laboratory of Complex Severe and Rare Diseases, Medical Research Center, Peking Union Medical College Hospital, Chinese Academy of Medical Sciences and Peking Union Medical College, Beijing, China; ^4^Department of Clinical Laboratory, Beijing Anzhen Hospital, Capital Medical University, Beijing, China

**Keywords:** carbapenemase, NDM, *Escherichia coli*, whole-genome sequence, antimicrobial resistance

In the original article, Starkova et al. (2021) was not cited in the article. The citation has now been inserted in **Introduction**, paragraph 2 and **Discussion**, paragraph 3.

Additionally, there was an error. Our article is the first research on NDM-29 in China rather than in Asia. Corrections have been made to **Highlights**, paragraph 1; **Introduction**, paragraph 2; **Discussion**, paragraph 1; **Discussion**, paragraph 3; and **Discussion**, paragraph 4.

The corrected paragraphs appear below.

**Highlights**, paragraph 1:

“- The first detailed report of the carbapenemase NDM-29 from *E. coli* and its genetic environment in China.

- A whole genome sequence analysis of the newly found plasmid pNC225-NDM-29 and the gene *bla*_NDM−29_.

- Complete functional verification experiments, including conjugation, transformation, cloning, and fitness cost.”

**Introduction**, paragraph 2:

“In a recent study, we discovered an NDM-29 carbapenemase- producing *E. coli* strain (19NC225), isolated from a patient's bile in 2019. The gene sequence of *bla*_NDM−29_ from a *Klebsiella pneumoniae* strain has been submitted to the NCBI database (NCBI), by researchers from Saint Petersburg, Russia (Starkova et al., 2021). Our study is the first detailed report of NDM-29 from *E. coli* in China, including genetic and phenotypic characterization, and confirms the potential threat of this new NDM-type carbapenemase to be a cause of extensively drug-resistant organism spread.”

**Discussion**, paragraph 1:

“Here we described a newly found carbapenemase, NDM-29, isolated from a clinical strain of *E. coli*. The success of conjugation from clinical isolation to *E. coli* EC600, *K. pneumoniae* ATCC 13883, and *K. pneumoniae* ATCC 700603 indicated the transferability of pNC225-NDM-29. The presence of the ISKpn19-based transposon where the region harboring *bla*_NDM−29_ is located suggested the risk of the spread of resistance caused by NDM-29. Meanwhile, no fitness cost was observed in the transconjugants/transformants containing plasmid pNC225-NDM-29, which demonstrated a limited burden from the transferable plasmid. However, the conjugation from donor to recipients (ATCC 13883, ATCC 700603, and DH5α) showed low efficiency, which may be due to high energy cost or multibarrier in recipients (Llosa et al., 2002). Although the growth curves did not show a significant impact of transferred plasmid on recipients, a limitation of growth curve that the competitive fitness of donor over recipients was not estimated and needs further study (Hanafi et al., 2016). Anyhow, the dissemination and adaptability of the NDM-29-harboring plasmid among clinical bacteria would be a threat to infectious control.”

**Discussion**, paragraph 3:

“Currently, the mutation of D130G has been reported in NDM- 14, and kinetic analysis indicated that NDM-14 has greater carbapenem resistance with a higher affinity for imipenem and meropenem (Zou et al., 2015). Based on our research, there is no difference between NDM-1 and NDM-29 in terms of susceptibility to carbapenems, which is in accordance with the findings of Starkova et al. (2021).”

**Discussion**, paragraph 4:

“In conclusion, we report the identification of a novel class B enzyme with carbapenemase activity, NDM-29, in a clinical *E. coli* isolate. The novel *bla*_NDM−29_ is first detected in China and was obtained from a MDR *E. coli* strain isolated from bile of a patient with biliary tract infection. The strain, containing two plasmids (pNC225-TEM1B and pNC225-NDM-29), belongs to ST1485 and O83:H42, showed homology with *E. coli* MS6198 from Australian (Hancock et al., 2017), which harbors *bla*_NDM−1_. The plasmid, pNC225-NDM-29, which encods *bla*_NDM−29_, exhibited 99% identity with six *bla*_NDM−1_-carrying plasmids, especially a IncN1 plasmid pNDM-BTR from an *E. coli* in urine specimen (99.96% identity and 100% coverage), and showed responsibility for the MDR phenotype.”

The authors apologize for these errors and state that this does not change the scientific conclusions of the article in any way. The original article has been updated.

## Publisher's Note

All claims expressed in this article are solely those of the authors and do not necessarily represent those of their affiliated organizations, or those of the publisher, the editors and the reviewers. Any product that may be evaluated in this article, or claim that may be made by its manufacturer, is not guaranteed or endorsed by the publisher.
