# Supplementary material for: Genetic and Phenotypic Characterization of the Novel Metallo-β-Lactamase NDM-29 From Escherichia coli
Source: Front Microbiol. 2021 Sep 29;12:743981. doi: 10.3389/fmicb.2021.743981 (PMC8511706; doi:10.3389/fmicb.2021.743981)
Supplement: Supplementary file 1 [file Table_1.DOCX]

**SUPPLEMENTARY MATERIAL**

Table S1.The primers used in this study.

| primer | 5'-3' | gene |
| --- | --- | --- |
| *bla*_NDM-29_-30box-F | CAAGCTTG-AAGCTTCTGTCGCACCTCATGTTTGAA | amplification of *bla*_NDM-29_ from NC225 |
| *bla*_NDM-29_-30box-R | AATCGC-GGATCCACGCCTCTGTCACATCGAAA |  |
| pHSG575-check-F | TCTGGAAGAAATAGCGCCC | sequencing of *bla*_NDM-29_ locus |
| pHSG575-check-R | CCTCCCAGAGCCTGATAAAA |  |

Table S2. The virulence genotype analysis of the sequences of chromosomes and plasmids of 19NC225

| Name**^*^** | Virulence genes |
| --- | --- |
| NC225-chr | *aec15, aec16, aec17, aec18, aec19, aec22, aec23, aec24, aec25, aec26, aec27/clpV, aec28, aec30, aec31, aec32, air, aslA, cfaABCD, cheABRWYZ, chuASTUVWXY, csgABCDEFG, eaeH, ecpABCDER, ehaB, elfAD, entABCDEFS, espL1, espR1, espX1, espX4, espY2, fepABCDEG, fes, fimABCDEFGHI, flgABCDEFGHIJKLN, flhABCDE, fliAEFGHIJKLMNOPQRSTYZ, flk, focX, gspCDEFGHIJKL, hcp, hcpA, hlyE/clyA, hofBC, ibeBC, icmF, kpsDM, matA/ecpR, matC/ecpB, matF/ecpE, motAB, ompA, papX, ppdD, sfaX, stgAB, tar/cheM, tia, upaG/ehaG, yagV/ecpE, yagW/ecpD, yagX/ecpC, yagY/ecpB, yagZ/ecpA, ycbFQRSTUV, yijP, ykgK/ecpR, APECO78_03970, APECO78_03975, APECO78_08685, CDCO157_0108, EC958_1421, EcE24377A_0237, ECH74115_1100, ECH74115_1101, ECO55CA74_05720, ECO55CA74_05725, ECO103_0224, ECO111_0230, ECP_0106, ECP_0107, ECP_0108, ECS88_3547, ECs0229, ECVR50_0238, ECVR50_0239, ECVR50_0242, ECVR50_0246, ECVR50_0247, ECVR50_0248, ECVR50_0249, ECVR50_0250, ECVR50_0257, ETEC_0103, ETEC_1007, O3M_20335, O3M_20925, O3M_20930, UMNK88_238, UMNK88_1093, UMNK88_1094, Z0260/tssF, Z0263, Z0265, Z1307, Z2200, Z2201, Z2203, Z2204, Z2205, Z2206* |
| pNC225-TEM1B | *aaiQR, iutA, iucABCD, sitABCD, ECVR50_3327* |
| pNC225-NDM-29 | none |

^*^Sequences of chromosomes end with the word “chr”; sequences of plasmids begin with the word “p”.

**
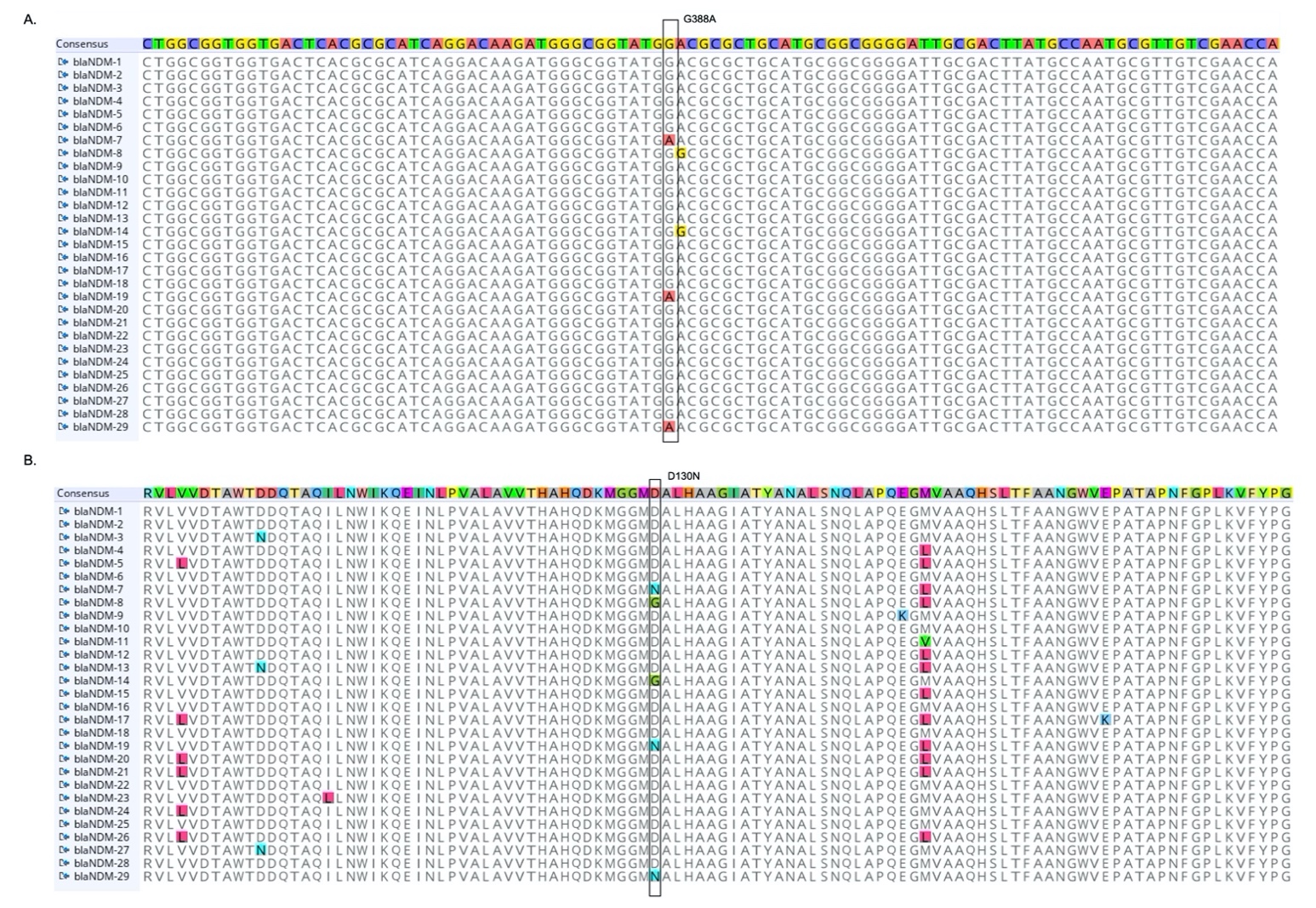
**

Figure S1. The sequence alignment results of *bla*_NDM-29_ gene and other *bla*_NDM_ variants. The difference alignment between *bla*_NDM_ variants is highlighted in color. The variation site in *bla*_NDM-29_ is indicated in the black box.
